# The possible influence of third-order shim coils on gradient–magnet interactions: an inter-field and inter-site study

**DOI:** 10.1007/s10334-023-01138-3

**Published:** 2024-01-10

**Authors:** Nicolas Boulant, Caroline Le Ster, Alexis Amadon, Guy Aubert, Alexander Beckett, Jean Belorgey, Cédric Bonnelye, Dario Bosch, David Otto Brunner, Guillaume Dilasser, Olivier Dubois, Philipp Ehses, David Feinberg, Sajjad Feizollah, Vincent Gras, Simon Gross, Quentin Guihard, Hervé Lannou, Denis Le Bihan, Franck Mauconduit, Frédéric Molinié, François Nunio, Klaas Pruessmann, Lionel Quettier, Klaus Scheffler, Tony Stöcker, Christine Tardif, Kamil Ugurbil, Alexandre Vignaud, An Vu, Xiaoping Wu

**Affiliations:** 1 CEA, CNRS, BAOBAB, NeuroSpin, University Paris-Saclay, 91191 Gif Sur Yvette Cedex, France; 2 CEA, Irfu, DACM, University Paris-Saclay, Gif Sur Yvette, France; 3 Brain Imaging Center and Helen Wills Neuroscience Institute, University of California, Berkeley, CA USA; 4 Advanced MRI Technologies, Sebastopol, CA USA; 5 CEA, Irfu, DIS, University Paris-Saclay, Gif Sur Yvette, France; 6 Department for Biomedical Magnetic Resonance, University of Tübingen, Tübingen, Germany; 7 High-Field MR Center, Max Planck Institute for Biological Cybernetics, Tübingen, Germany; 8 Skope MRT, Zurich, Switzerland; 9 Center for Neurogenerative Diseases, Bonn, Germany; 10 Montreal Neurological Institute-Hospital, McGill University, Montreal, QC Canada; 11 ETH Zürich and University of Zürich, Zurich, Switzerland; 12 Center for Magnetic Resonance Research, University of Minnesota, Minneapolis, MN USA; 13 University of California, San Francisco, CA USA; 14 San Francisco VA Health Care System, San Francisco, CA USA

**Keywords:** Vibrations, Shim coils, Field monitoring, Gradient, Magnet interactions, Ultra-high field

## Abstract

**Objective:**

To assess the possible influence of third-order shim coils on the behavior of the gradient field and in gradient–magnet interactions at 7 T and above.

**Materials and methods:**

Gradient impulse response function measurements were performed at 5 sites spanning field strengths from 7 to 11.7 T, all of them sharing the same exact whole-body gradient coil design. Mechanical fixation and boundary conditions of the gradient coil were altered in several ways at one site to study the impact of mechanical coupling with the magnet on the field perturbations. Vibrations, power deposition in the He bath, and field dynamics were characterized at 11.7 T with the third-order shim coils connected and disconnected inside the Faraday cage.

**Results:**

For the same whole-body gradient coil design, all measurements differed greatly based on the third-order shim coil configuration (connected or not). Vibrations and gradient transfer function peaks could be affected by a factor of 2 or more, depending on the resonances. Disconnecting the third-order shim coils at 11.7 T also suppressed almost completely power deposition peaks at some frequencies.

**Discussion:**

Third-order shim coil configurations can have major impact in gradient–magnet interactions with consequences on potential hardware damage, magnet heating, and image quality going beyond EPI acquisitions.

**Supplementary Information:**

The online version contains supplementary material available at 10.1007/s10334-023-01138-3.

## Introduction

A steady increase of image resolution in MRI has been occurring since its birth thanks to advancement in hardware including RF coils [1], gradient coils [2–4], their associated amplifiers [5, 6], and magnets. After a leading effort by the Center for Magnetic Resonance Research to perform in vivo imaging at 10.5 T [7], now the highest magnetic field available for human studies is provided by a 11.7 T whole-body scanner, called Iseult, located in Saclay France [8], with a promise to reach higher Signal-to-Noise Ratio (SNR) [9, 10] and, thus, higher spatio-temporal resolutions. To make the most of these ultra-high fields, powerful gradients (i.e., high slew rate and gradient strength) are needed. Yet, higher magnetic fields and higher gradient strength and slew rates lead to stronger forces and mechanical vibrations possibly reaching local accelerations on the order of 1000 g [8]. These vibrations can couple via magneto-mechanical interaction to the gradient and shim coils, cryostat and cryoshields, dissipating energy in the He bath. Lorentz forces in the cryostat likewise can be induced by the imperfectly shielded time-dependent magnetic field which can generate eddy currents and vibrations, again depositing energy by Joule effect. Given that magneto-mechanical coupling is multi-physical and highly complex [11], it is essential to take into account both mechanical and electromagnetic aspects when predicting power deposition in the He bath [8, 12]. Decoupling mechanically the gradient coil from the magnet and shielding the gradient field help to reduce gradient–magnet interactions and, thus, He boil-off.

Another important concern related to the strong mechanical vibrations of the gradient coil is field disturbances and associated image artifacts. In [8], physical displacements of the gradient coil on the order of a couple of hundreds of µm could be determined on a whole-body gradient coil at 11.7 T only at certain critical frequencies and at maximum gradient strength compatible with the maximum slew rate. Unless high resolution images are targeted, because the spectrum of an MR sequence in general has its energy spread over a relatively large frequency range, these displacements at first sight do not pose a serious difficulty to faithful spatial encoding. In the presence of a static magnetic field, vibrations, however, can engender eddy currents in the different conducting structures by Faraday’s law. Exponentially decaying eddy currents arising from electromagnetism alone are usually corrected by signal demodulation and pre-emphasis [13]. Vibration-induced currents can also induce field oscillations that are more difficult to compensate, manifesting themselves as peaks and dips in the Gradient Transfer Function (GTF), i.e., the Fourier transform of the Gradient Impulse Response Function (GIRF) [14], and causing image artifacts. The vibration of the gradient coil also induces an electromotive force (emf) in its circuit and a current, thereby changing the effective impedance of the gradient coil seen by the Gradient Power Amplifier (GPA) [15]. The same vibration likewise can induce field distortions through coupling with shim coils or/and magnet. The scanner calibration steps of tuning-up the GPA and its Proportional–Integral–Derivative (PID) controller aim at canceling the perturbations and maintaining the nominal current. In [8], the insertion of a lead tube surrounding the gradient coil was intended to screen gradient–magnet interactions and protect the Iseult magnet [12]. Its presence, however, led to large peaks in the GTF of the Z gradient axis and accordingly to strong ghosting artifacts in EPI when the corresponding resonances were excited with certain echo spacings. The corresponding artifacts on images acquired on a phantom can be found in [8]. Removing the lead tube cleaned significantly the spectrum, reducing or eliminating nearly all peaks except for an important one remaining at around 1350 Hz. This peak, which lies outside of the traditional forbidden zones advised by the gradient coil manufacturer, still appeared to cause problems for gradient waveform fidelity, including in anatomical imaging when gradient spoilers are employed. Due to the lack of accurate modeling methods, the purpose of this work was to elucidate this problem by conducting an inter-site study where characterization of the same type of whole-body gradient coils was performed via GIRF measurements [14, 16] at different field strengths (7 T, 9.4 T, 10.5 T, and 11.7 T) and with different magnets, electronics and setups. Furthermore, because mechanical coupling between the vibrating gradient coil and the magnet (e.g., bore tube, cryoshields) can lead to more eddy currents and field perturbations, different boundary or mechanical decoupling conditions were examined on Iseult to investigate their impact on the field response, taking advantage of the fact that this magnet could be ramped up and down.

## Materials and methods

### Theoretical background: sensitivity of the imaging gradients on the details of the GTF

**Fig. 1:**
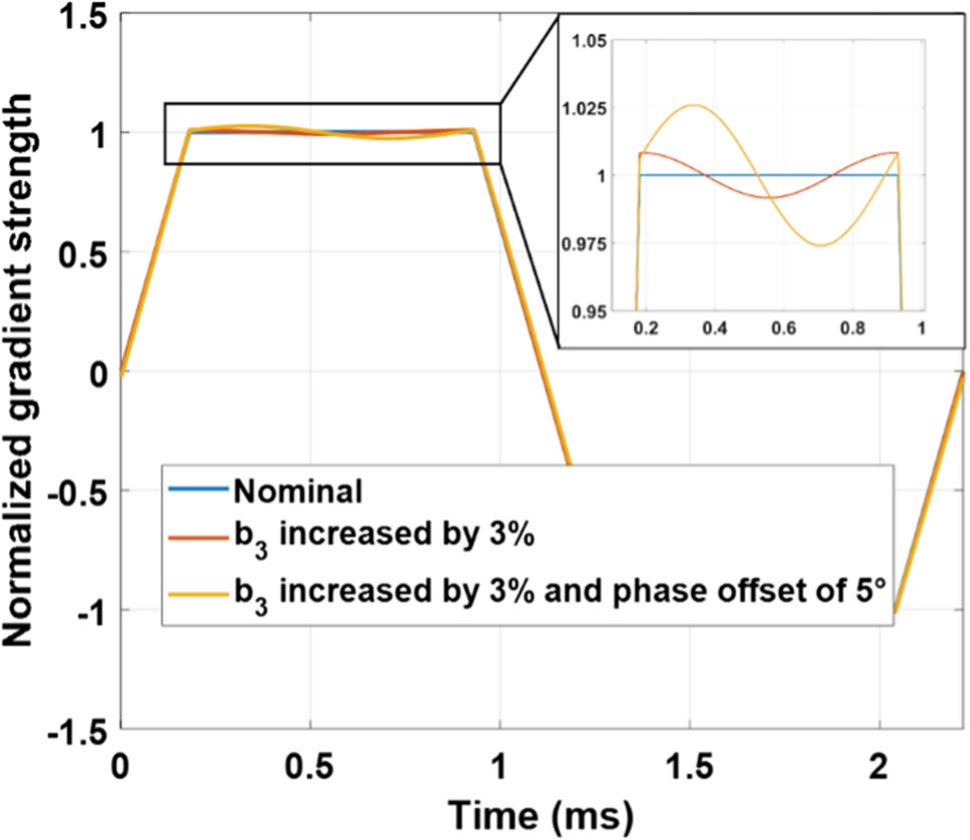
Theoretical impact of GTF imperfections on an EPI waveform. Two distorted waveforms were generated by increasing the third harmonics of the nominal waveform (blue) by 3% in magnitude without phase offset (red) and with 5° phase offset (yellow)

Deviations in the GTF from a unit response can have strong consequences on imaging [8, 14]. An infinitely long EPI waveform *s*(*t*) with ramp and plateau durations t_1_ and t_2_, respectively (Echo-Spacing ES = 2*t*_1_ + *t*_2_) and gradient strength *G*, can be decomposed into the following Fourier series $s\left(t\right)=G\sum_{n=\mathrm{1,3},5\dots }^{+\infty }{b}_{n}{\text{sin}}\left(2\pi n\nu t\right)$, where ${b}_{n}=\frac{4(2{t}_{1}+{t}_{2})}{{n}^{2}{\pi }^{2}{t}_{1}}{\text{sin}}\left(\frac{n\pi {t}_{1}}{2{t}_{1}+{t}_{2}}\right)=\frac{2}{{n}^{2}{\pi }^{2}{t}_{1}\nu }{\text{sin}}(2n\pi {t}_{1}\nu )$ and $\nu =\frac{1}{2{\text{ES}}}$. The 1350 Hz mechanical resonance therefore for instance can be excited with EPI by setting ES = 0.37 ms or ES = 1.11 ms, which correspond to the first (*n* = 1) and third (*n* = 3) harmonics of the EPI train. A simple exercise highlights how sensitive the gradient waveforms employed in MRI are with respect to small imperfections in the GTF. Figure 1 reports a nominal EPI waveform (normalized by *G*) generated with 1000 Fourier coefficients and the same waveform whose third harmonics has been amplified by only 3% and dephased either by 0° or 5°. Such small deviations already lead to relatively strong perturbations of the gradient waveform and, thus, of the *k*-space trajectory. Importantly, the results also show the importance of the phase response. For small phase offsets $\varphi$ of only the *n*th harmonics, the relative maximum difference between the nominal gradient waveform and the distorted one is ${\varphi b}_{n}$. Furthermore, we shall show in the manuscript that the transient time to reach a stationary condition is long compared to a typical read-out EPI train [17]. As a result, there is a risk that the phase calibration pre-scans [18] used by some vendors only see the beginning of the transient regime and therefore lack accuracy to correct all echoes when field disturbances evolve along the EPI train.

### Characterization of the resonance at 1350 Hz in the Iseult nominal configuration

As shown in [8], the GTF measured on Iseult at 11.7 T revealed a strong resonance at 1350 Hz on the *Z* gradient axis in the absence of the lead tube. To better understand its origin, GIRFs were also acquired on a Classic Magnetom 7 T system (VB17 software, passively shielded whole-body Agilent magnet) at the same site to characterize the amplification of the field disturbances versus magnetic field as well as identify potential differences between setups. A GIRF measurement at CMRR at 10.5 T was also performed to obtain an additional data point between the two field strengths. The three scanners are equipped with the same whole-body SC72 gradient coil (weight = 900 kg, length = 1.59 m, inner diameter = 64 cm, outer diameter = 81 cm, inductance = 870/845/820 (X/Y/Z) µH, DC resistance = 140/130/135 (*X*/*Y*/*Z*) mΩ, maximum gradient strength = 70 mT/m and maximum slew rate = 200 mT/m/ms) commercialized by Siemens Healthineers (Siemens Healthcare, Erlangen, Germany). The measurements consisted of characterizing the GIRF using a clip-on field camera (Skope MRT, Zürich, Switzerland), using the methodology described in [8]. To complement the GTF spectra, EPI and gradient spoiler waveform measurements were also performed to illustrate in the time domain the problems encountered. For simplicity, in this work we focus on the most problematic Z gradient axis where the peaks and dips in the GTF spectrum were the strongest. Additional information about the other gradient axes is provided in the online resource material.

### Investigations on mechanical coupling

**Fig. 2:**
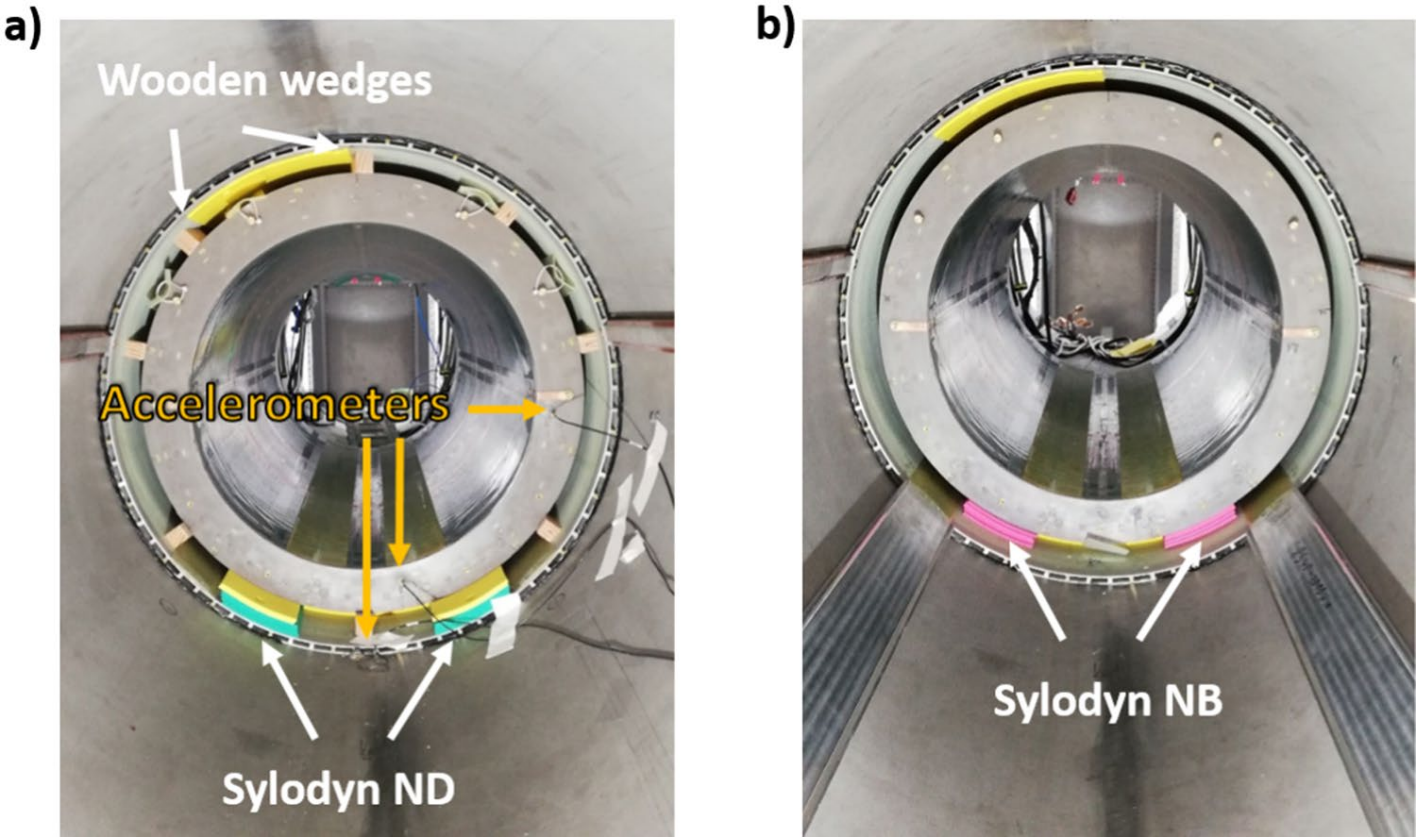
Iseult experimental setups showing the SC72 whole-body gradient coil inside the magnet bore. Mechanical coupling between the gradient coil and the magnet was modified by inserting wooden wedges (**a**) or by changing the Sylodyn material from ND (green) to the NB (pink) type (**b**). The accelerometers are also shown in (**a**), those on the right and at the bottom used to measure accelerations in the left–right and antero-posterior directions, respectively

In [8], measurements versus field strength with accelerometers revealed in the nominal configuration of Iseult that interestingly vibrations at 1350 Hz on the Z axis reached a plateau at about 8 T up to 11.7 T, due to an apparent increased damping. To study the impact of mechanical coupling between the gradient coil and the magnet on the field response, several scenarios were investigated. First, we measured on Iseult’s 11.7 T magnet ramped to 7 T the GTF without the iron passive shim tray to establish the presence of a possible vibration of magnetic material via magneto-mechanical coupling. The gradient coil also rests on so-called Sylodyn pads (Getzner, Bürs, Austria) in the magnet bore which are meant to mechanically decouple the gradient coil from the magnet. To alter the mechanical coupling and study its impact on the field dynamics, the Sylodyn pads initially of type ND (green) were replaced by the NB ones (pink) with different mechanical properties (including stiffness), and the GIRF measurements were repeated. Measurement of the vibrations was performed with gradient sweeps at 1 mT/m and over the 0–3 kHz frequency range in 2 min, using mono-axial accelerometers (Brüel & Kjaër, Naerum, Denmark) located on the gradient coil and the magnet bore to verify whether mechanical coupling was truly affected. Finally we attempted this time to increase mechanical coupling by inserting wooden wedges between the circular perimeter of the gradient coil and the bore, at both extremities. Pictures of the setups are provided in Fig. 2. A mechanical simulation using Ansys (Ansys, Canonsburg, PA, USA) also was conducted to identify the vibration eigenmodes of a cylinder (free boundary conditions for simplicity) with same dimensions of the SC72 gradient coil and with multilayered glass–epoxy properties [19], to gain physical insight into the particular 1350 Hz mode. After confirming mechanical coupling changes, measurement of the power deposition in the Iseult He bath was performed around the 1350 Hz resonance at 11.7 T to investigate the impact of this coupling, and GIRF measurements were conducted for all these setups. The Iseult superconducting coil is immersed in a pressurized He bath, which is cooled down to 1.8 K. To reach and maintain this temperature, a cryo facility pumps the He gas. The speed of the pumping unit varies depending on the power deposition to maintain a constant temperature. However, the correlation between this speed and the power delivered is not trivial. As a result, it was decided to maintain the pumping speed constant, thus the cooling power, and impose a constant 10W power with a heater embedded in the He bath. The 10W power then is compensated by the cooling unit to reach equilibrium. When the temperature of the bath is disturbed by gradient activity, the power of the heater adjusts automatically to regulate temperature and maintain it constant. Therefore, one can deduce the power deposition with the gradient fields from the change of power dissipated by the heater. While the 7000 l volume of helium and the 1.8 K temperature in the Iseult magnet provide a large safety buffer for MR operation, on the other hand it requires time-consuming gradient frequency sweeps (< 100 Hz/h) to cover a large frequency range and obtain the desired helium boil-off spectra. For this reason, this test campaign focused on the frequency intervals of interest where power deposition peaks could be determined in some previous work [8] and the frequency sweep rate employed was 50 Hz/h.

### Investigations on GPA type and third-order shim coils

Measurements were also performed at other sites equipped with different magnets and with different fields, scanner generations, GPA and third-order shim configurations (connected or disconnected). Because vibrations are unique to a gradient coil type and its geometry, measurements were performed at all sites with the same whole-body SC72 gradient coil. All sites also were equipped with the same Skope field camera technology. The driving currents were also measured at the output of the GPA 90/22 of Iseult at 0 T and 7 T, and on a classic Magnetom 7 T (GPA XXL) scanner using an oscilloscope with current sensors. Two different GPA types were tested on Iseult at 11.7 T: (1) the so-called Siemens 90/22 (2250 V, 950 A max) initially installed (nominal configuration), and (2) the Siemens XXL (2000 V, 650 A max). Although we could not get direct information about the GPA control bandwidths, they can be indirectly inferred from the full-width at half maximum of the GTF spectra, revealing on the order of 40 kHz bandwidth for both GPA types. Finally, GTF measurements on Iseult at 11.7 T, on a Terra 7 T and on the 10.5 T Magnetom were performed with the third-order shim coils connected versus disconnected at the filter plate on the gradient side, inside the Faraday cage.

Table 1 summarizes the different setups. All gradient SC72 coils here incorporated third-order shim coils (four terms: Z3, Z2X, Z2Y, Z(X2–Y2)), located between the primary and the shielding layers. The shim amplifiers had maximum voltage/current of 20 V/20 A for the Terra-based series. A “No” in the table simply indicates that third-order shim coils were either disconnected for the tests either by choice or because a corresponding shim power amplifier was not available.

**Table 1:** List of the different setups and sites where GIRF measurements with a field camera were carried out

Scanner	Field strength (T)	GPA	Third-order shim connected	Software	Site
Iseult	11.7	90/22	Y/N	VE12	CEA, NeuroSpin, Saclay, France
Magnetom	10.5	XXL	Y/N	VE12	CMRR, UMN, MN, USA
Magnetom	9.4	XXL	N	VE12	MPI, Tuebingen, Germany
Iseult	7	90/22	Y	VE12	CEA, NeuroSpin, Saclay, France
Terra	7	90/22	Y/N	VE12	McGill, Montreal, Canada
Terra 7 T Plus	7	XXL	N	VE12	SFVA, San Francisco, CA, USA
Classic Magnetom 7 T	7	Boosted-XXL	N	VB17	CEA, NeuroSpin, Saclay, France

All scanners were equipped with the whole-body SC72 gradient coil. The boosted GPA XXL on the classic Magnetom 7 T allows reaching 100 mT/m and 200 mT/m/ms on the SC72 whole-body gradient coil

## Results

### GTF at 11.7 T (Iseult), 10.5 T (CMRR), and on the classic 7 T Magnetom

**Fig. 3:**
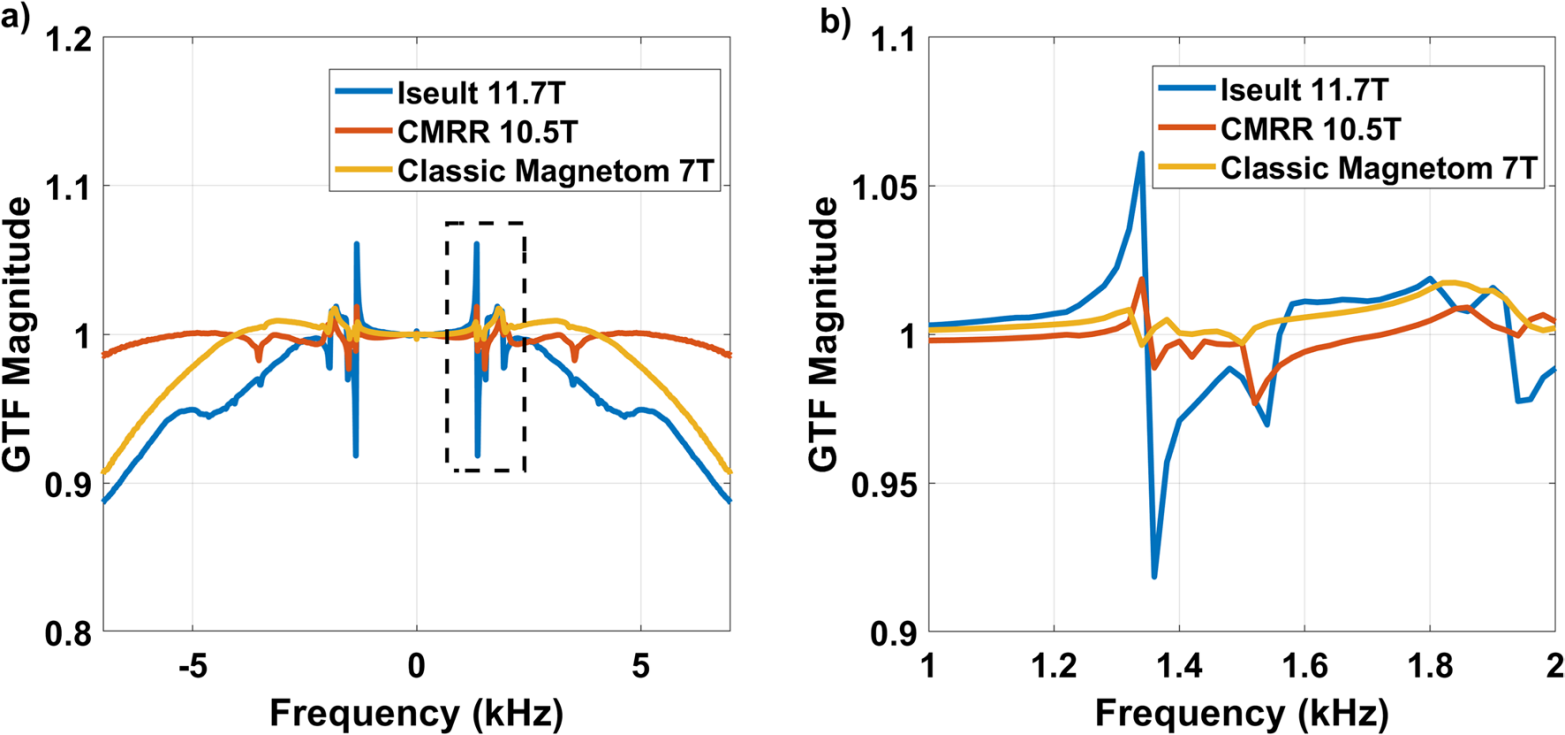
Illustration of the 1350 Hz strong resonance on the gradient Z axis observed on Iseult at 11.7 T. **a** GTF spectra (magnitude) over the − 7 and + 7 kHz range. **b** Zoom on the 1–2 kHz range showing the main resonance. Comparisons are provided with a measurement at 10.5 T (CMRR) and 7 T (Classic Magnetom at NeuroSpin)

Figure 3 presents the GTF acquired on a classic 7 T Magnetom as well as 11.7 T initially on Iseult (other characteristics listed in Table 1) and at 10.5 T at CMRR (in its nominal configuration, i.e., with third-order shim coils disconnected). The 11.7 T result was the one reported in [8], i.e., without the lead tube and with the third-order shim coils connected (nominal configuration). The data revealed at first a supra-linear amplification of the peak at 1350 Hz when going from 10.5 to 11.7 T. Although an intensification of the peak versus field strength was to be expected because of increased gradient–magnet interactions, such drastic amplification suggested that other factors were important.

### EPI waveform measurements

**Fig. 4:**
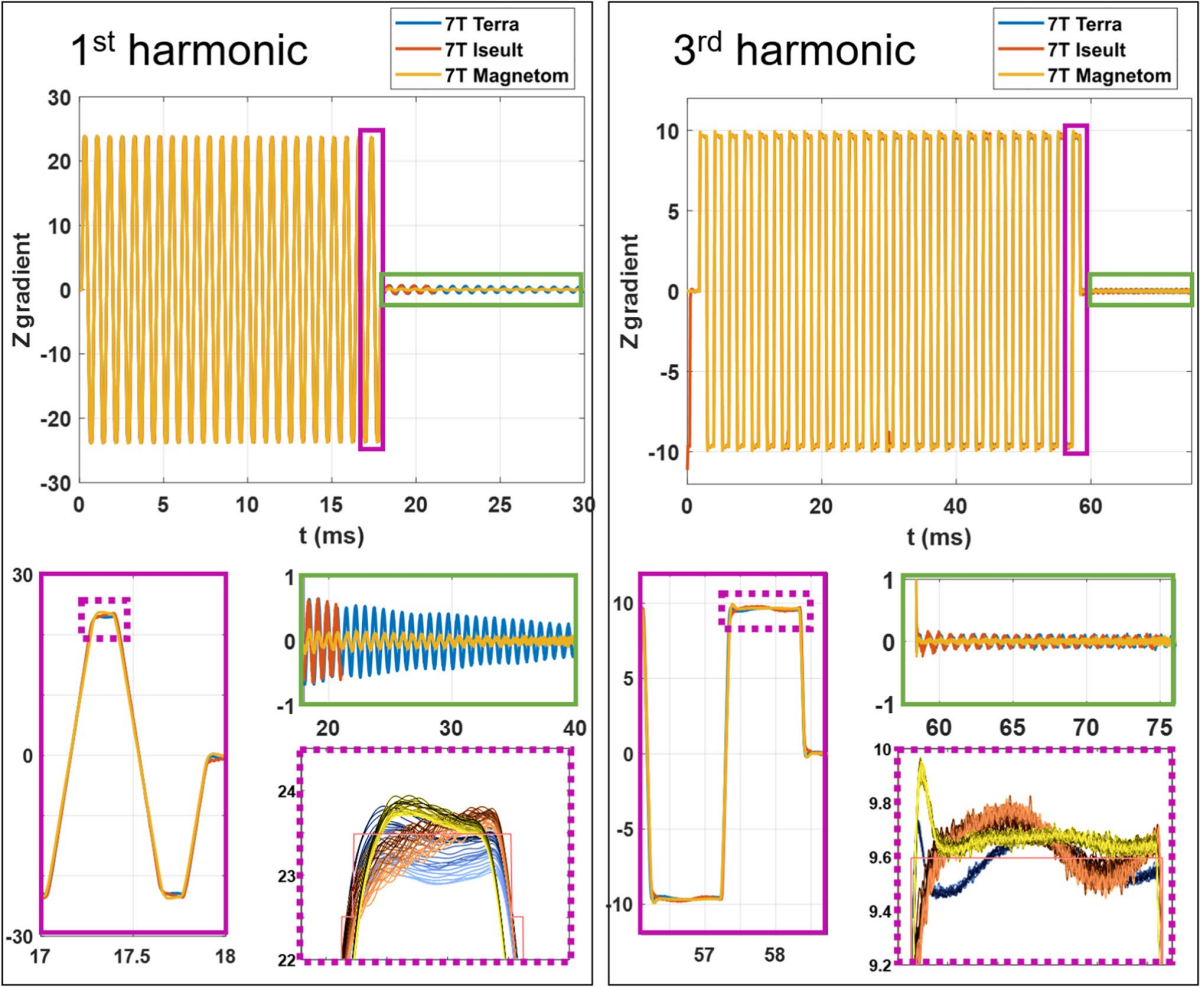
EPI field perturbations observed at 7 T when exciting the 1350 Hz resonance on the Z gradient axis. Left: 1st harmonic (ES = 0.37 ms) excitation, right: third harmonic (ES = 1.11 ms) excitation. A zoom on the plateaus (purple boxes) and on the oscillations after the EPI train (green boxes) is provided in each case, with the same color coding. The plateaus along the EPI train are superposed at the bottom. The yellow–black, orange–brown, light and dark blue plots in the zoom correspond to the Magnetom, Iseult, and Terra measurements. The darker the color for each group, the earlier the plateau occurs in the EPI train. Units are in mT/m in all figures. On the left, the red curve of the oscillation after the EPI train was interrupted to leave visible the Terra data

Some analysis of field measurements was performed in the time domain to get more insight about the effects induced by the peaks and dips visible in the GTF spectra. Figure 4 reports EPI waveforms measured with a field camera at 7 T on Iseult, at 7 T on a Terra and on the classic 7 T Magnetom when first (ES = 0.37 ms) and third (ES = 1.11 ms) harmonics of the EPI train excite the 1350 Hz mechanical resonance. One can see that the vibrations distort the EPI plateaus and that gradient oscillations can even persist after the train. Of course, because there is no data acquisition in that period, the latter are not strictly problematic for faithful spatial encoding but instead illustrate better the impact of vibrations on the field, because eddy currents alone would yield exponentially decaying fields [13]. A zoom into the superposed measurements on the gradient plateaus also reveals a transient behavior hypothesized to be due to mechanical damping, because of the order of magnitude of the time-constants, which yields a characteristic time comparable to the duration of the EPI train, making phase correction pre-scans [17, 18] possibly inaccurate. The oscillations after the train here were comparable on 7 T Iseult and 7 T Terra, while the classic 7 T Magnetom yielded smaller oscillations. The decline of the amplitude of the EPI plateaus along the train with the first harmonic is also more severe on the Terra and Iseult than on the classic Magnetom, at the same field strength. This suggests similarities between the two former systems, 7 T Iseult and 7 T Terra, regarding this particular problem. Despite an overshoot at the beginning of the EPI plateaus with the classic Magnetom 7 T, field perturbations with the third harmonic excitation oscillate more with the 7 T Terra and 7 T Iseult around the center of k-space.

### Mechanical coupling alterations

**Fig. 5:**
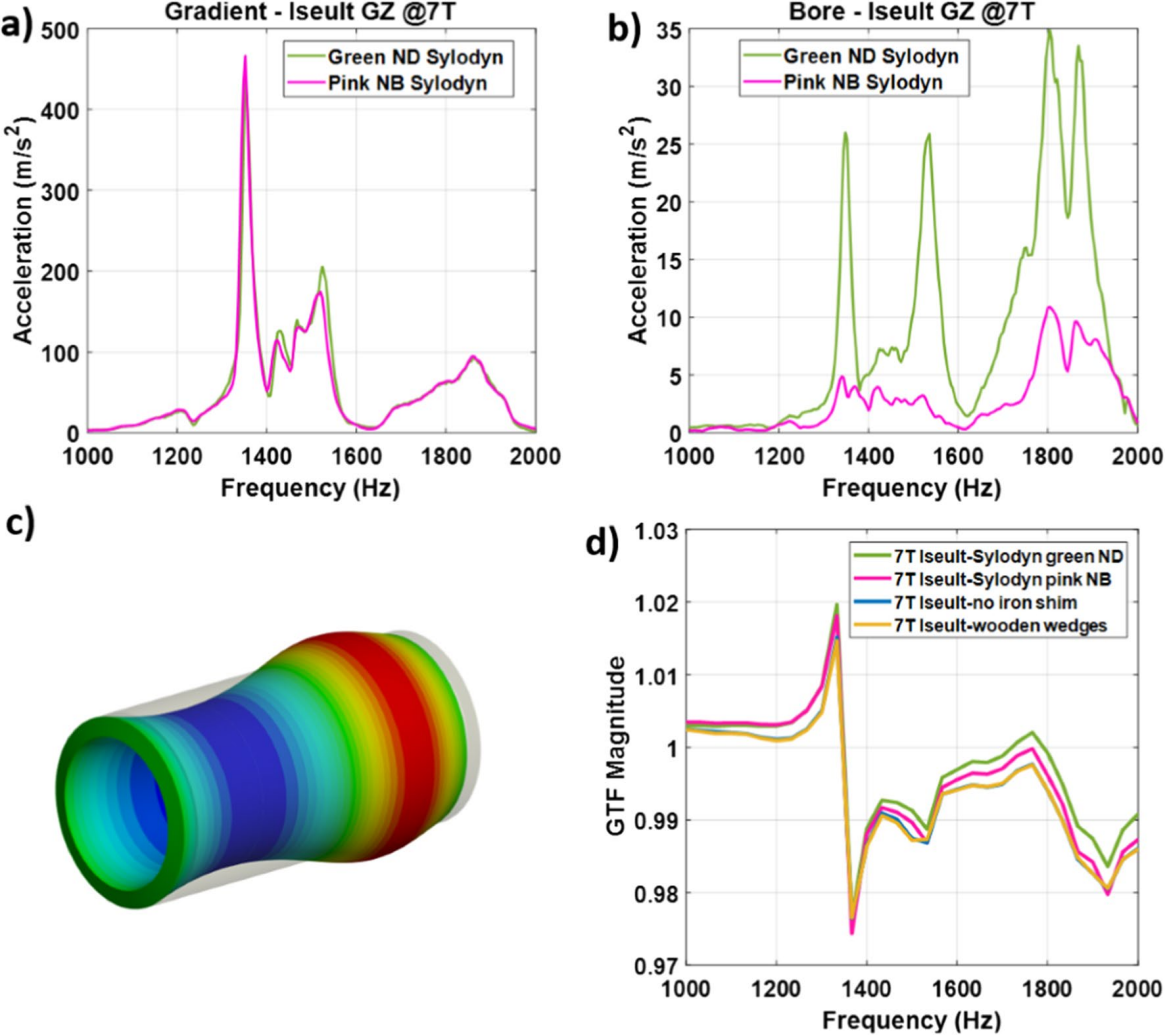
Mechanical coupling alteration results on Iseult operated at 7 T. Subplots (**a**) and (**b**) show the accelerations measured on the gradient coil and the bore tube, respectively, with green (ND) and pink (NB) Sylodyn pads. Subplot (**c**) reports the deformation returned from a mechanical simulation as a breathing mode being the closest resonance matching the experimental data. Subplot (**d**) reports the magnitude of the GTF measurement around the 1350 Hz resonance in the various conditions where mechanical coupling was altered, showing little differences

Figure 5 reports the accelerations versus frequency measured on the SC72 gradient coil and the Iseult magnet bore at 7 T when pulsing on the gradient *Z* axis at 1 mT/m and with the ND (green) and NB (pink) Sylodyn pads. The mechanical resonance at 1350 Hz is clearly visible. Interestingly, the vibration spectra of the gradient coil are quasi-identical in the two scenarios while coupling to the bore has been significantly reduced using the NB (pink) Sylodyn. Figure 5d shows the GTF magnitude around the main resonance in the different configurations, where very little difference was found. The result of the mechanical simulation identified the closest resonance at 1415 Hz, in reasonable agreement with the experimental data, and is illustrated in Figure 5c. The mode is labeled (0,2) indicating 0 and 2 half-wavelengths in the circumferential and axial directions. It is called a breathing mode because displacements perpendicular to the *z* axis maintain cylindrical symmetry. The data shows that mechanical coupling was significantly altered but yet the field response remained highly similar. Insertion of wooden wedges and removing the passive shim tray also had little effect on the field response. The reader should note that a tune-up of the PID controller of the GPA was repeated systematically in each condition so that small differences can be the result of the slight variations in regulator tuning. In the end, the data convincingly shows that the mechanical coupling between the gradient coil and the magnet was not responsible for the main peak intensity in the GTF spectrum located at 1350 Hz in Fig. 3.

### GPA and third-order shim coil influence results

**Fig. 6:**
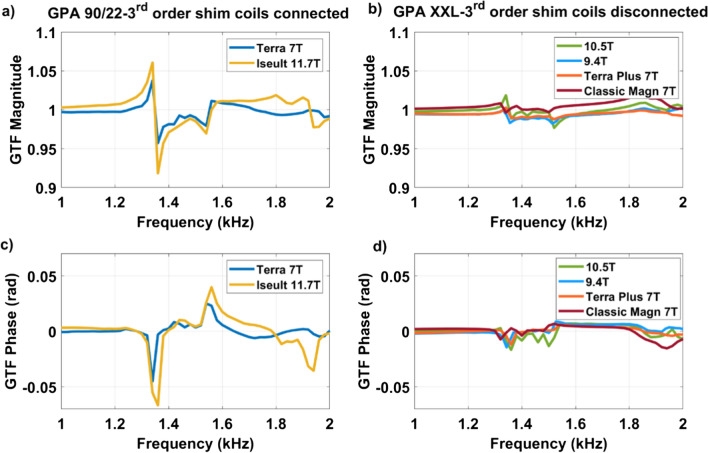
Self-term Z GTF measurements for the SC72 whole-body gradient coils when driven by the GPA 90/22 (third-order shim connected) versus GPA XXL (third-order shim disconnected) at various field strengths. **a**, **b** Magnitude and **c**, **d** phase in the GTFs are provided

Figure 6 reports the magnitude and phase of the GTF on Iseult at 11.7 T, 10.5 T (CMRR), 9.4 T (MPI, Tuebingen), 7 T Terra (McGill, Montreal), 7 T Terra Plus (SFVA), and 7 T classic Magnetom (CEA NeuroSpin). Data were separated into two groups for clarity, i.e., with the GPA XXL with no third-order shim coils connected and the GPA 90/22 with third-order shim coils connected, the nominal configurations. The data are plotted on the same scale to emphasize the differences between the two scenarios. There is clearly a distinct behavior where for instance the 1350 Hz peak at 7 T with the GPA 90/22 (Terra) with connected third-order shim is higher than the one obtained at 9.4 T and 10.5 T with the GPA XXL with third-order shim disconnected, despite a lower field strength, especially in the phase response. For the same GPA XXL, the peak tends to become moderately higher with field strength (from 7 to 9.4 T and 10.5 T). Overall, the data of Fig. 6, thus, highlighted two potential key differences, i.e., GPA and third-order shim configurations, between the setups that could affect the results.

### Current measurements on Iseult

**Fig. 7:**
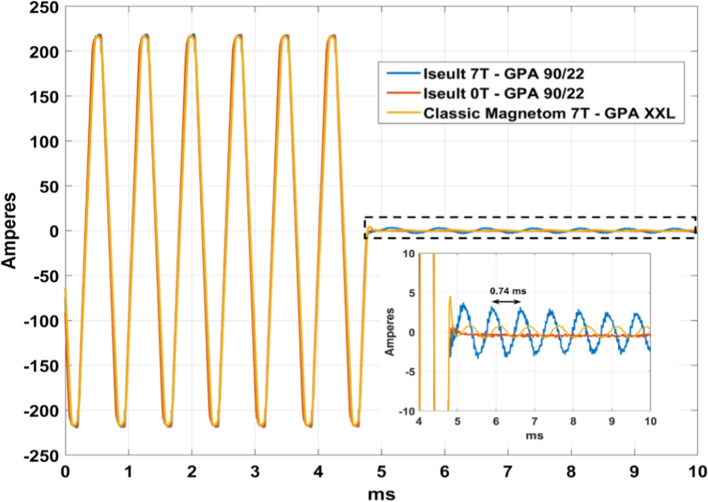
Current measurements on the GPA during an EPI sequence with ES = 0.37 ms. The absence of oscillations at 0 T confirmed the influence of the vibrations on the current. The GPA XXL (classic Magnetom) with no third-order shim coils connected led to smaller oscillations than the GPA 90/22 with third-order shim coils connected when both used at the same field strength of 7 T

The results of Fig. 6 show a different gradient field behavior between two setups involving different GPAs and third-order shim connections, as characterized by the field camera technology. To gain further insight, current measurements were performed directly on the GPAs on Iseult at 0 T (GPA 90/22), Iseult at 7 T (GPA 90/22) and on the classic Magnetom 7 T (GPA XXL). Results are displayed in Fig. 7. At 0 T, there are no vibrations and, therefore, there are no oscillations at the end of the EPI train. The currents, however, oscillate in the presence of the main field and are larger with the GPA 90/22 with connection to the third-order shim coil versus the GPA XXL with no connection, respectively, for the same field strength. With an echo-spacing of 0.37 ms, the main frequency of the EPI waveform drives the 1350 Hz mechanical resonance with deformation pictured in Fig. 5c. At the end of the train, the system is released and vibrates predominantly at the same frequency, given this eigenmode initial condition. The data, therefore, are consistent with the gradient field oscillations shown in Fig. 4.

### Impact of third-order shim coils

**Fig. 8:**
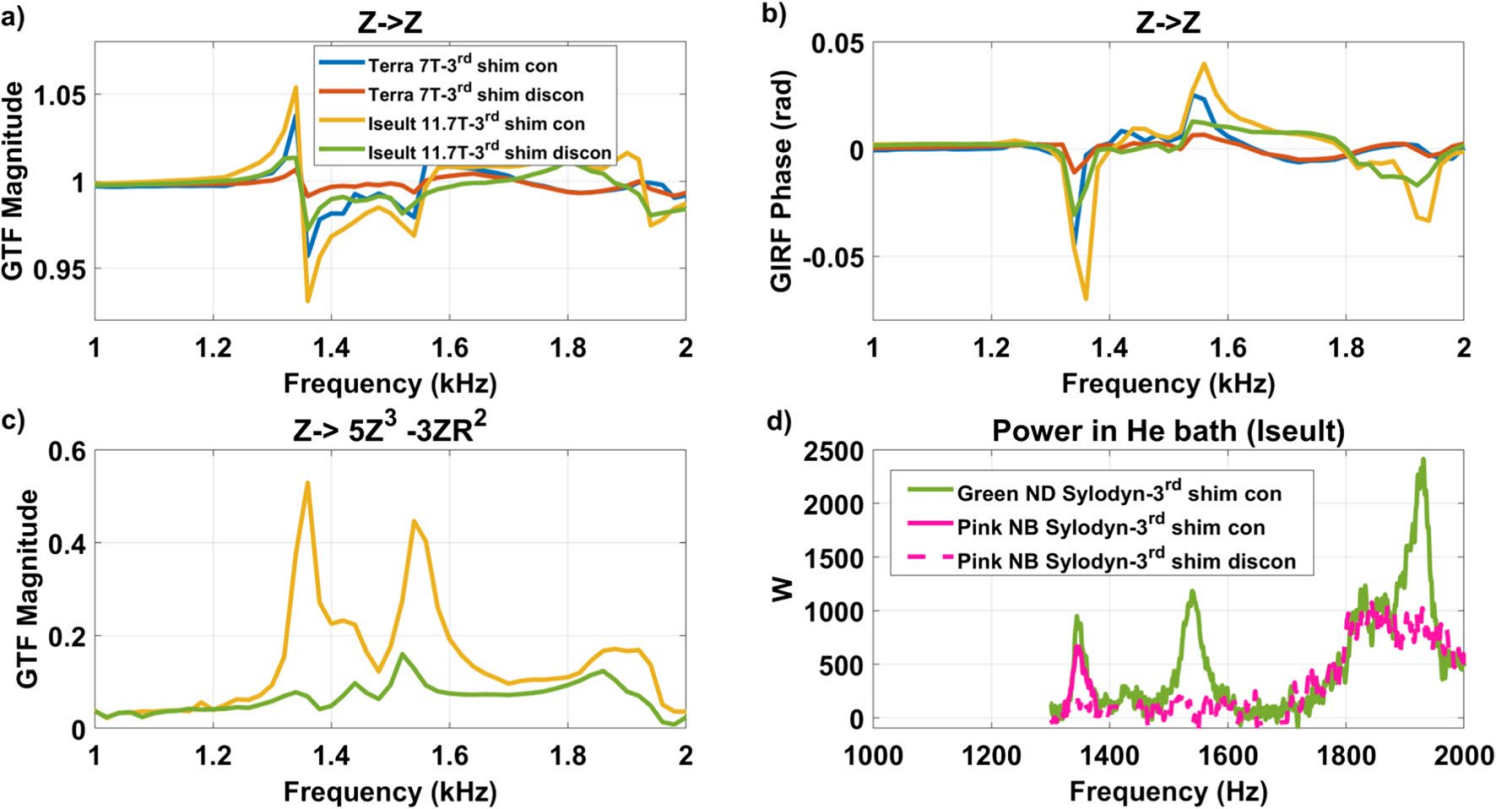
Impact of the third-order shim coils. GTF **a** magnitude and **b** phase self-term responses are shown for the Terra 7 T and Iseult 11.7 T systems (third-order shim coils connected and disconnected). Subplot **c** shows the most important third-order spherical harmonics (*R*^2^ = *X*^2^ + *Y*^2^ + *Z*.^2^) response measured with a dynamic field camera on Iseult at 11.7 T with the third-order shim coils connected vs. disconnected (same color legend). Subplot **d** reports the power deposition measurement in the He bath of Iseult at 11.7 T between 1300 and 2000 Hz (renormalized for 70 mT/m)

The results of Fig. 6 indicated that both the GPA type and the third-order shim coils configuration could possibly play a role. To attempt to solve the puzzle, a GPA XXL was, therefore, installed and configured on Iseult and the GIRF measurements were repeated at 11.7 T. But little differences were found. The separation made in Fig. 6 (left: GPA 90/22, right: GPA XXL), thereby simply reflected the chronology of events and current knowledge when the experiments were carried out. Therefore, after showing that the GPA type barely had any influence, the GPA 90/22 was reinstalled with the third-order shim connected or disconnected to the filter plate inside the Faraday cage on the gradient side. The results are reported in Fig. 8. Connection to the third-order shim cabinet, even with its amplifier disabled, had major impact on the GTF (results shown both for Terra 7 T and Iseult 11.7 T). A similar measurement was performed with the second-order shim coils disconnected but little differences were observed. A Skope dynamic field camera was also employed to reconstruct the dynamic third-order spherical harmonics fields for better accuracy and SNR at 11.7 T, when driving the *Z* gradient axis. The most dominant Z3 (⇒5Z^3^-3ZR^2^) term is shown in Fig. 8c which reveals matching peaks with the self-term, suggesting current circulation in the corresponding shim coil at the resonance frequencies when the latter is connected to the filter plate. Figure 8d also reports the measurement of the power deposition (renormalized for 70 mT/m, assuming power deposition is proportional to the square of the gradient amplitude, as verified experimentally before) in the Iseult He bath at 11.7 T versus frequency for the gradient *Z* axis between 1300 and 2000 Hz: with green (ND) Sylodyn (third-order shim coils connected) and pink (NB) Sylodyn (third-order shim coils connected and disconnected). The pink (NB) Sylodyn had a non-negligible but still relatively smaller impact on the results. This shows that mechanical coupling played a role, yet smaller, and that the interaction mostly responsible for the power deposition is electromagnetic, the 1350 Hz mechanical resonance yet remaining the root cause. The cryogenic measurement with the pink (NB) Sylodyn pads and with the third-order shim coils connected was conducted only over the frequency range of most interest for this work (1300–1400 Hz), because of the stress it engenders on the gradient cables and gradient coil.

**Fig. 9:**
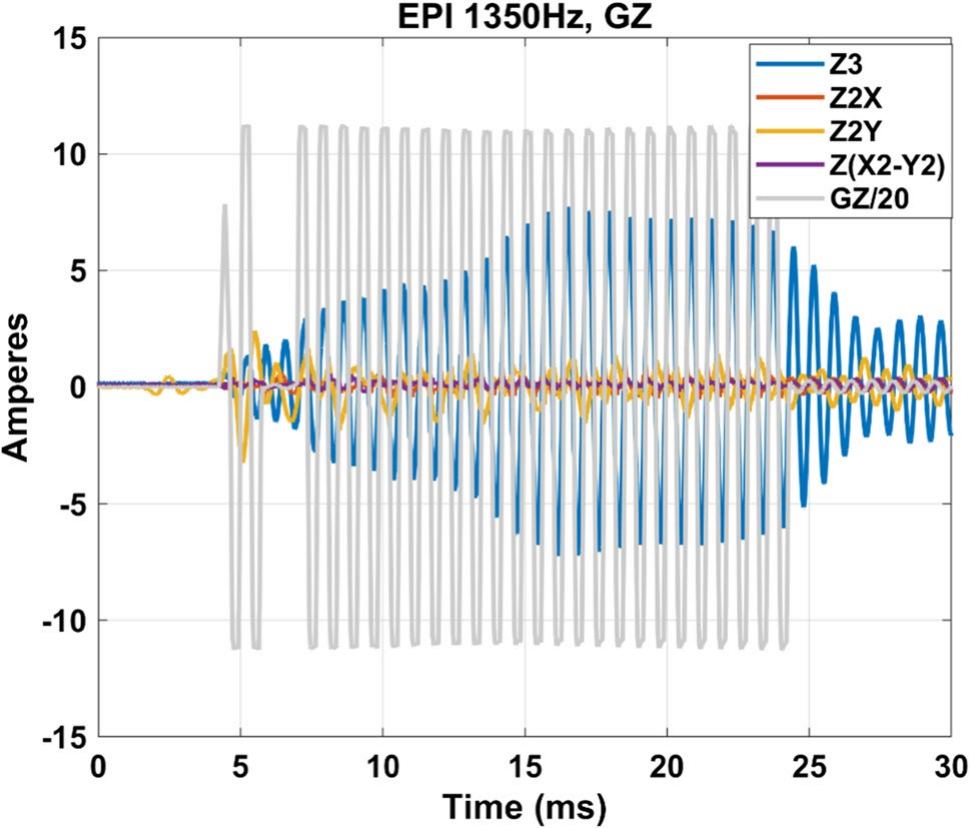
Currents flowing in the *Z* gradient and in the third-order shim coils when the 1350 Hz mechanical resonance is excited. The current in the *Z* gradient coil has been scaled down by a factor of 20 to superpose it to the other waveforms

Figure 8c suggests current flowing in the third-order shim coils when driving the *Z* gradient coil at some particular frequencies. To further confirm this behavior, current measurements through each individual third-order shim coil were repeated with current clamps on Iseult at 11.7 T with an EPI waveform with H–F read-out axis and ES = 0.37 ms (1/2ES = 1350 Hz), when the shim coils are connected fully, i.e., all the way to their shim amplifiers. The results are plotted in Fig. 9 and reveal indeed currents oscillating at the same frequency. One can observe again a transient regime. In such instance, therefore, the 3-line reference method before the EPI train cannot capture well the field perturbations occurring in the center of k-space.

**Fig. 10:**
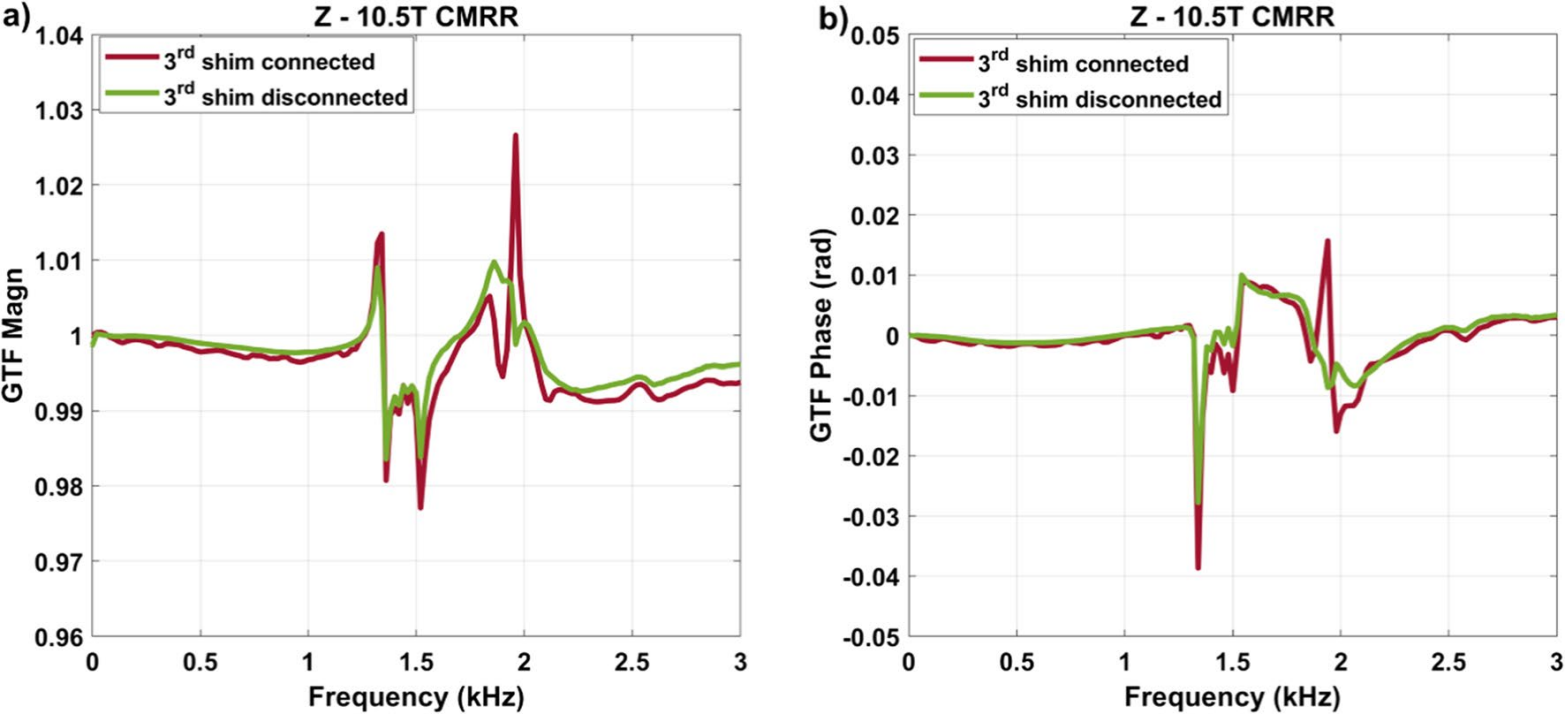
GTF measurements (*Z* self-term) on the SC72 gradient coil at 10.5 T at CMRR with third-order shim coils connected versus disconnected. **a** Magnitude and **b** phases are shown

Figure 10 reports the GTF measurement performed at 10.5 T (CMRR) with and without the third-order shim coils connected. The data again demonstrate a significantly different behavior between the two scenarios. Nevertheless, although clearly a difference remains at 1350 Hz, one can see another significant difference at 1950 Hz. Although other differences may not have been identified yet, this is possibly a result of the shim filters which are of a different generation on this system (classic Magnetom versus Terra), which can affect the current flow.

**Fig. 11:**
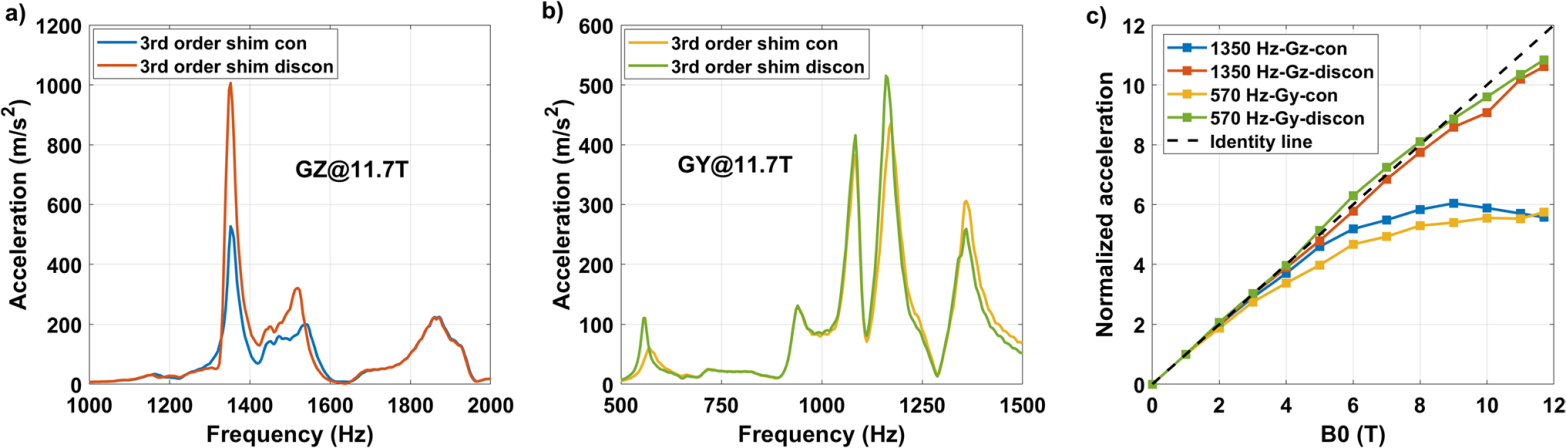
SC72 gradient coil vibration results versus third-order shim coil configuration (connected or disconnected). Results for **a** GZ and **b** GY are provided for 1 mT/m excitation and at 11.7 T. The normalized accelerations versus *B*_0_ are reported in (**c**) for the *Z* breathing (1350 Hz) and the *Y* banana (570 Hz) modes, revealing distinctly different behaviors with respect to the third-order shim coil configuration

Lastly, to gain further understanding a vibration measurement of the gradient coil was performed on Iseult when ramping from 0 to 11.7 T at every Tesla, again with the third-order shim coils connected or disconnected, when pulsing at 1 mT/m on the *Y* or *Z* gradient axes. The results are presented in Fig. 11. Interestingly, the gradient coil vibrates more at 1350 Hz when the third-order shim coils are disconnected. The same behavior can be observed for the banana mode at 570 Hz on the *Y* gradient axis. When normalized to the response characterized at 1 T, the vibration versus *B*_0_ yields distinctly different behaviors reported in Fig. 11c. When the third-order shim coils are disconnected, vibrations at those frequencies grow linearly with *B*_0_. When they are connected, they reach a plateau at around 8 T as in [8, 20].

### Spoiler waveform measurements

**Fig. 12:**
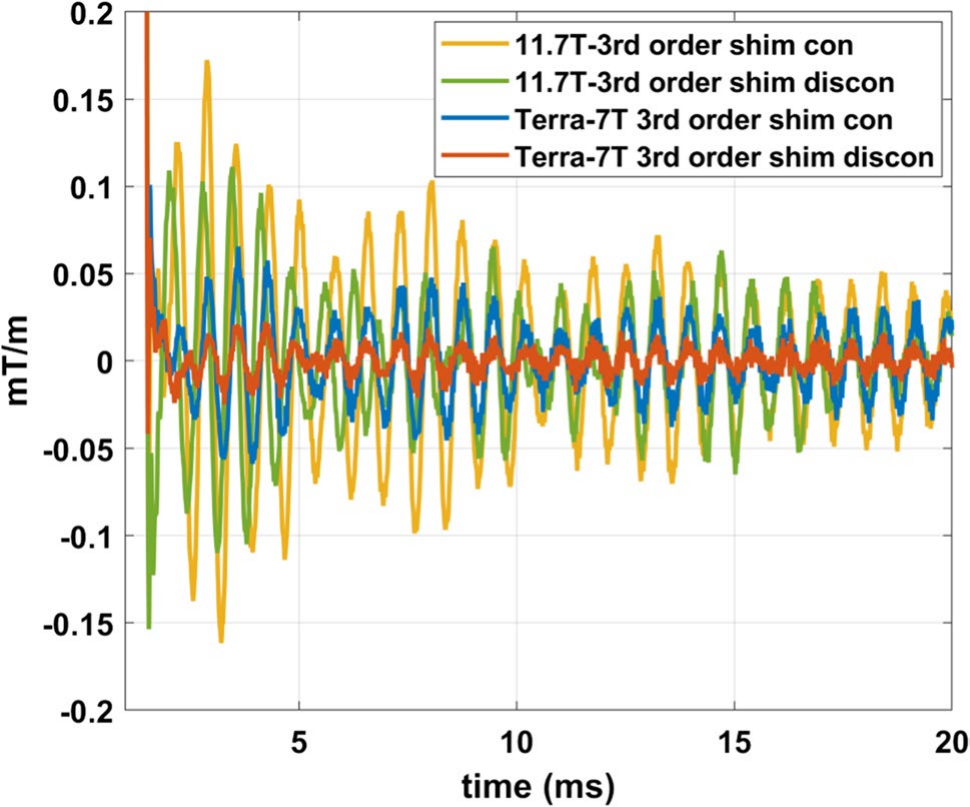
Gradient oscillations measured after a gradient spoiler of 40 mT/m. The main oscillation frequency observed in all four SC72 whole-body gradient scenarios (i.e., Iseult 11.7 T and Terra 7 T with third-order shim coils connected, Iseult 11.7 T and Terra 7 T with third-order shim coils disconnected) was 1350 Hz

Figure 12 shows field monitoring measurements of gradient spoilers typically used in anatomical imaging, e.g., the MPRAGE, with Iseult 11.7 T and Terra 7 T (third-order shim coils connected and disconnected). Gradient oscillations persist after the spoiler event ending at 1 ms. The gradient field oscillation roughly corresponds to a 100 Hz field variation offset at 5 cm from isocenter in the *Z* direction at 7 T on the Terra. The oscillations are reduced by disconnecting the third-order shim coils on both scanners.

## Discussion

We have shown that mechanical vibrations of the whole-body gradient coil type investigated in this work and induced by Lorentz forces lead to resonances in the GTF which are highly dependent on the third-order shim configuration, i.e., third-order shim coils connected or disconnected at the filter plate inside the Faraday cage. The influence of the mechanical coupling between the gradient coil and the magnet was ruled out as the cause of the strong peaks and dips in the GTF of Iseult by altering drastically the boundary conditions with different Sylodyn pads and wooden wedges (Fig. 5). Luckily, removing the iron shim tray did not have an impact on the field response either so that it could be put back in place to meet the static field homogeneity specifications. The precise physical explanation about the influence of the third-order shim coils is not well understood yet. The interaction is complex and we lack information about their design, which is proprietary. But connection of the third-order shim coils to the filter plate and all the way to the shim amplifiers presumably simply provides a path for the current to flow (Figs. 8c, 9), possibly resonate and interact back with the gradient coil by mutual inductance [15]. Given the strong mechanical resonance identified with accelerometers (Fig. 5), it is clear that the root cause of the problem is vibrations (see also current measurements in Fig. 7). But how it couples to the shim coils and how exactly it influences the gradient field for the moment remains to be determined, while the circuit details and the shim filters could play a role too, as suggested by the results obtained on the 10.5 T scanner in Fig. 10**.** The vibration data shown in Fig. 11c reveals growing interactions with B_0_ between the gradient and shim coils when current can circulate in the latter, effectively yielding greater vibration damping. Although counterintuitive at first sight, more vibration of the gradient coil when the third-order shim coils were disconnected did not lead to more field disturbances visible in the GTF spectra. The increased vibration damping versus *B*_0_ with third-order shim coils connected, therefore, was a manifestation of increased interactions in fact detrimental to imaging. One additional finding of great importance in this work is the impact of the third-order shim coils on power deposition in the He bath. It is, therefore, assumed that coupling to this term generates a field distribution of particular influence on the power deposition. One possible explanation under consideration is a reduction of the gradient shielding efficiency engendered by the vibrations and induced currents, or simply current circulation in the third-order shim coils induced by vibrations and resulting in unshielded fringe fields leading to more power deposition. Despite the apparent benefits of the lead tube on power deposition reported in [8] on the *Z* gradient axis, with the new data reported here, one can reasonably assume even more efficient screening and, thus, reduced He boil-off thanks to that tube when the third-order shim coils are disconnected, given the disappearance of the power deposition peak at 1350 Hz (Fig. 8d) that would likely occur in the presence of the lead tube as well. The additional peaks and dips in the GTF spectrum in its presence, however, still make the lead tube hardly compatible with MRI operation. Due to the lack of appropriate connectors, the influence of the installed 4 different third-order shim terms could not be tested individually in detail. The GTF data of Fig. 8c (and online resource), however, clearly show the dominance of the Z3 term, as further confirmed by the current measurements in Fig. 9. Because the 1350 Hz power deposition peak was not predicted by our model including the gradient coil, the He vessel and the cryoshields [12], the result emphasizes the importance of taking into account this possible coupling phenomenon as well as the gradient coil mechanical resonances for accurate prediction. In this work, disconnecting the third-order shim coils indeed decreased significantly the power deposition in the He bath (Fig. 8d). But without further data, this phenomenon cannot so easily be extrapolated to other magnet designs. Given the drastic differences observed, great caution is, therefore, advised when first experiments are conducted on new design magnets with gradient coils embedding third-order shim coils.

Different scanner tune-ups at different dates could have an impact on the GIRF response and the GTF. It was found, however, that repeating the tune-up of the PID had little effect compared to the differences between setups. The GTF characterized on the Terra in Montreal is also quite similar to the one in Würzburg reported in [21], suggesting reasonable inter-site reproducibility for the same exact configuration. Struck initially by the differences obtained with different GPAs (Fig. 6), measurements with both the XXL and 90/22 were performed on Iseult. But it was then established finally that the third-order shim configuration was the parameter having most impact across the different tests and that the GPA in fact had little influence. From the measurement results of Fig. 7, it appears that an undesired current circulates in the gradient coil which the GPA does not compensate for perfectly. More general details on GPAs for instance can be found in [5, 6, 15, 22–24].

Emphasis in this study was put on the gradient *Z* axis, for which the phenomenon was the most severe. Figure 11 (and online resource material) yet reports some results on the *Y* axis with the same observation about the behavior versus connection to the third-order shim coils. The online resource material also shows coupling of the *X* and *Y* axes to different third-order shim coils which likewise could be confirmed with GTF measurements. The field disturbances yet are not as strong, especially on the phase, than on the Z axis. As motivated above, this can be a consequence of circuit geometry, frequency, displacements, and vibration mode shapes. In addition, the main peaks of the *Y* axis with the SC72 in Fig. 11 are in the traditionally forbidden intervals advised by the coil manufacturer. Yet again, they could be excited with higher-order harmonics of EPI shapes. Within the range of validity of the formalism, for a linear time-invariant system [25] the GTF returns the distortion of the Fourier components of the gradient waveform. The theory and experimental data demonstrated that the interactions at 7 T and above can be strong enough to significantly alter gradient shapes.

Given eddy currents and strong vibrations, MR users are used to dealing with imperfections in EPI sequences. Shifting slightly the echo-spacing is common practice and shall avoid exciting mechanical resonances and help minimizing ghosting artifacts. This, however, becomes more problematic at higher field strengths such as at 11.7 T if the peaks become more intense and if the frequency regions where significant artifacts occur become broader (see Fig. 3). To avoid damage or ghosting, the SC72 gradient coil normally has two forbidden bands: 500–600 Hz and 950–1250 Hz. Including 1350 Hz with the first- and third-order harmonics of an EPI train, thus, would lead to extending these bands to ~ 400–600 Hz and ~ 950–1400 Hz, which would be problematic for MR operation as more echo spacings would be forbidden. Field monitoring of course is one way to deal with the gradient field imperfections [26, 27] by taking them into account in the reconstruction. Pre-emphasis by inversion of the linear time-invariant system model is another option although precautions need to be made to make sure the returned input waveforms are feasible [28]. Both approaches yet have great impact on the workflow and pre-characterization of the system requires a high degree of time invariance of the system [25]. In any case, field monitoring cannot mitigate potential damage of the gradient coil or elevated heating in the cryostat. Furthermore, viewing this phenomenon as just an EPI problem would be a mistake since field perturbations shall be induced whenever a gradient sequence excites the strong resonances. As an example, Fig. 12 showed gradient fields generated by gradient spoilers commonly used in anatomical imaging. Following the gradient events, oscillations again caused by vibrations persist and might interfere with RF pulses, especially those designed for parallel transmission where sub-pulses are often concatenated and phase coherence among them is required [29]. This phenomenon also has been reported to be problematic in spectroscopy for the identification and quantitation of metabolite resonances [30]. The oscillations indeed can persist beyond 10 ms and are consistent with independent observations in [21]. Increasing the TR accordingly to leave space between the gradient spoiler and the next RF pulse would be unacceptable for some anatomical sequences, e.g., MPRAGE, where the duration of the read-out train cannot be too long to preserve a good point spread function and contrast. Caution also should remain in EPI sequences where spoilers likewise can be used. One possible way to tackle the problem is to slow down the current ramps to decrease the high frequency content of the gradient shapes, still at the expense of a longer TR. Choosing more favorable gradient axes is also another way to mitigate the problem but may also be not possible for instance with rotating diffusion encoding gradients. For all the reasons mentioned above, mitigating the problem at the source is worth pursuing. Yet we regret that at this stage we cannot fully explain the phenomena observed (vibrations, power deposition and GTF). Going further would require more detailed information about the shim filters, the shim coils design and possibly the GPA, which is proprietary. It is our hope that with the data presented, gradient and shim coil designers could take this problem more into consideration. To our knowledge, such drastic effects caused by the coupling of the gradient coil investigated here with the third-order shim coils was unknown and it is clear that such coupling can represent a source of inter-site variability.

To investigate the problem, measurements performed at different sites was one of the strategies employed in this work. This approach was also used in the past to estimate inter-site differences in brain imaging [31]. Each site in the group having a unique setup with respect to magnet, electronics, software, GPA, shim configuration, with comparisons we were able to eliminate variables and converge toward the component responsible for the significantly increased peak response in the GTF from 7 to 11.7 T, when third-order shim coils were connected. All partners having a Skope field camera, the same GIRF measurements with the same sequence and methodology were consistent and could be carried out at all sites. Keeping also the same identical setup on Iseult while changing the field strength or the boundary conditions was invaluable and key to obtaining an understanding that mechanical coupling between the gradient coil and the magnet was not responsible for the field behavior observed in the GTF. Finally, the comparisons made in this study led to the conclusion that the large peak and dip initially observed at 1350 Hz on Iseult 11.7 T in its nominal configuration [8] was not the result of an inevitable law of nature we had to live with but rather a problem that could be mitigated. For better gradient waveform fidelity, less field perturbations and less magnet heating, in the current setup it has been decided for now to leave unplugged the third-order shim coils on Iseult.

## Conclusion

We have identified with inter-site field monitoring measurements a strong field distortion problem caused by an interaction between the gradient and third-order shim coils, whose implications go beyond EPI and can also have an impact on anatomical imaging with gradient spoilers. Field strength amplification of the phenomenon from 7 to 11.7 T had much less impact. It is advised that this problem be considered at an early stage when exploring on other ultra-high field MR scanners operated at a field strength above 7 T.

### Supplementary Information

Below is the link to the electronic supplementary material.

Supplementary file1 (DOCX 1143 KB)

## Data Availability

Data is available upon reasonable request.
