# Supplementary material for: The possible influence of third-order shim coils on gradient–magnet interactions: an inter-field and inter-site study
Source: MAGMA. 2024 Jan 10;37(2):169–83. doi: 10.1007/s10334-023-01138-3 (PMC10995016; doi:10.1007/s10334-023-01138-3)
Supplement: Supplementary file 1 — Supplementary file1 (DOCX 1143 KB) [file 10334_2023_1138_MOESM1_ESM.docx]

[Magnetic Resonance Materials in Physics, Biology and Medicine](https://www.springer.com/journal/10334)

**The possible influence of third order shim coils on gradient-magnet interactions: an inter-field and inter-site study**

N. Boulant^1*+^, C. Le Ster^1^, A. Amadon^1^, G. Aubert^2^, A. Beckett^3,4^, J. Belorgey^5^, C. Bonnelye^1^, D. Bosch^6,7^, D. O. Brunner^8^, G. Dilasser^2^, O. Dubois^5^, P. Ehses^9^, D. Feinberg^3,4^, S. Feizollah^10^, V. Gras^1^, S. Gross^8^, Q. Guihard^5^, H. Lannou^2^, D. Le Bihan^1^, F. Mauconduit^1^, F. Molinié^5^, F. Nunio^5^, K. Pruessmann^11^, L. Quettier^2^, K. Scheffler^6,7^, T. Stöcker^9^, C. Tardif^10^, K. Ugurbil^12^, A. Vignaud^1^, A. Vu^13,14^, X. Wu^12^

^1^University Paris-Saclay, CEA, CNRS, BAOBAB, NeuroSpin, Gif sur Yvette, France

^2^University Paris-Saclay, CEA, Irfu, DACM, Gif sur Yvette, France

^3^Brain imaging center and Helen Wills Neuroscience institute, University of California, Berkeley, California, USA

^4^Advanced MRI technologies, Sebastopol, California, USA

^5^University Paris-Saclay, CEA, Irfu, DIS, Gif sur Yvette, France

^6^Department for biomedical Magnetic Resonance, University of Tübingen, Tübingen, Germany

^7^High-field MR center, Max Planck Institute for biological cybernetics, Tübingen, Germany

^8^Skope MRT, Zürich, Switzerland

^9^Center for Neurogenerative Diseases, Bonn, Germany

^10^Montreal Neurological Institute-Hospital, McGill University, Montreal, Quebec, Canada

^11^ETH Zürich and University of Zürich, Zürich, Switzerland

^12^Center for Magnetic Resonance Research, University of Minnesota, Minneapolis, Minnesota, USA

^13^University of California, San Francisco, California, USA

^14^San Francisco VA Health Care System, San Francisco, CA, USA

*Corresponding author

^+^Besides the first two, authors are listed in alphabetical order

Email: [nicolas.boulant@cea.fr](mailto:nicolas.boulant@cea.fr)

# Y self-terms of the GTF on Iseult at 11.7T, 10.5T at CMRR and on a 7T Terra with and without connection of the 3^rd^ order shim coils


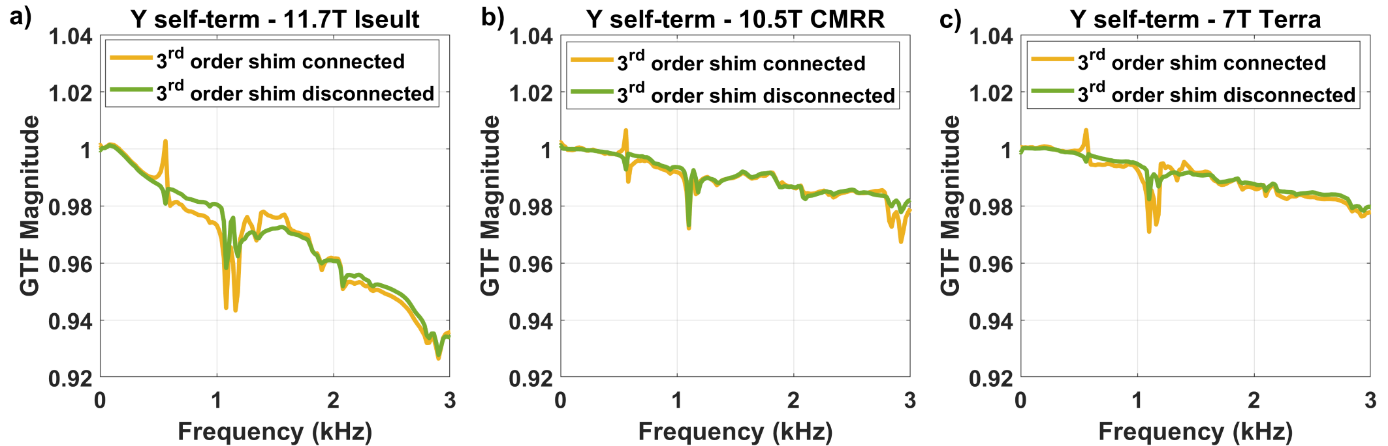


***Fig S1****. First order (Y) self-terms of the gradient transfer function measured with and without connection of the 3^rd^ order shim coils. Results are shown for a) Iseult 11.7T, b) CMRR 10.5T and c) Terra 7T. On all three systems, a noticeable difference is visible at around 560 Hz (banana mode). The Iseult and Terra have the same shim filters and likewise have different peak magnitudes at around 1150 Hz in the two configurations. The 10.5T may be equipped with different shim filters which may lead to a larger difference at 2950 Hz.*

# Self-to-third order spherical harmonics field cross-terms measured at 11.7T on Iseult with a dynamic field camera

**
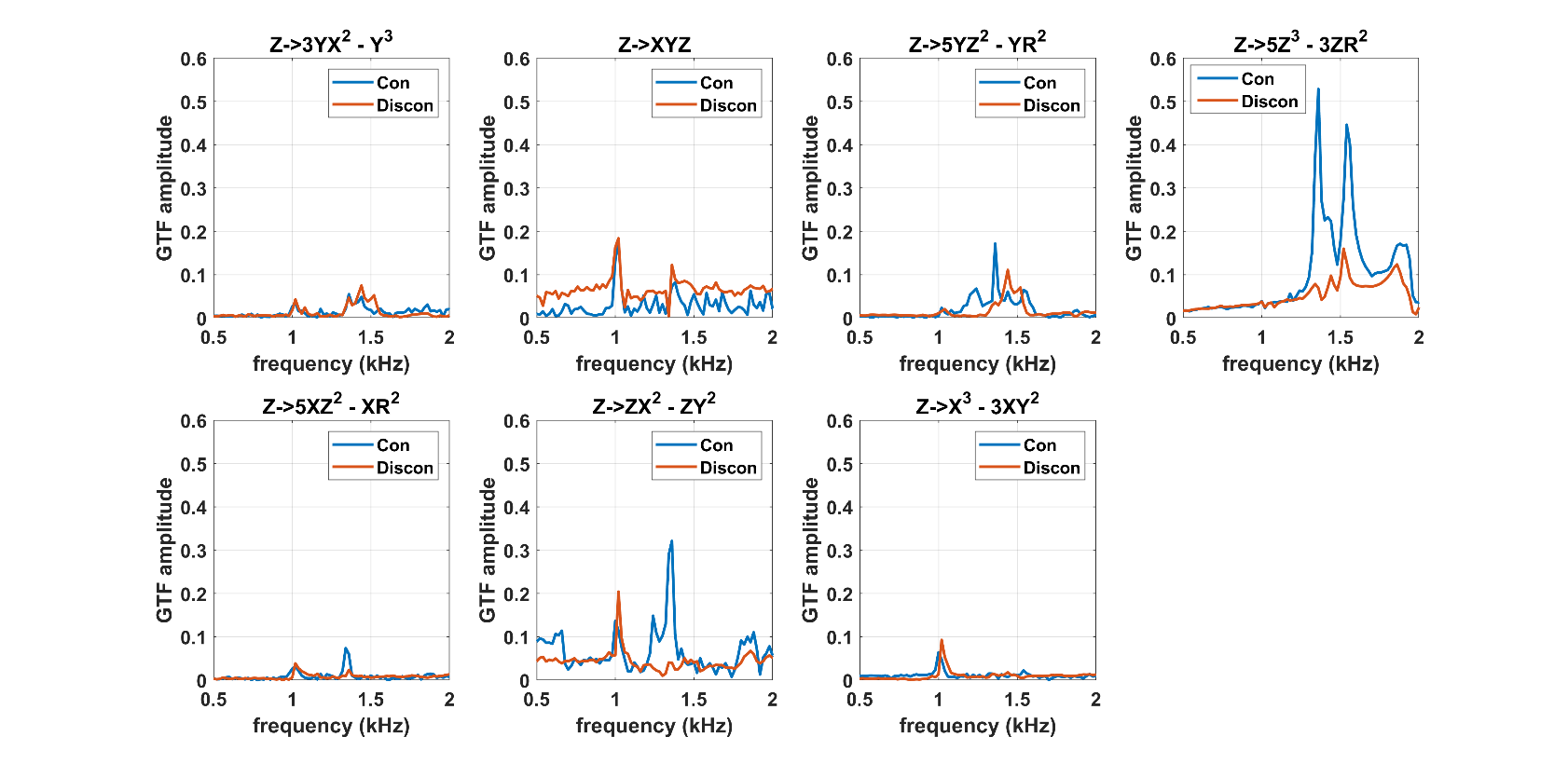
**

***Fig S2****. First to third order field cross-terms measured at 11.7T with a dynamic field camera with the 3^rd^ order shim coils connected and disconnected, when pulsing on the Z gradient axis.*

**
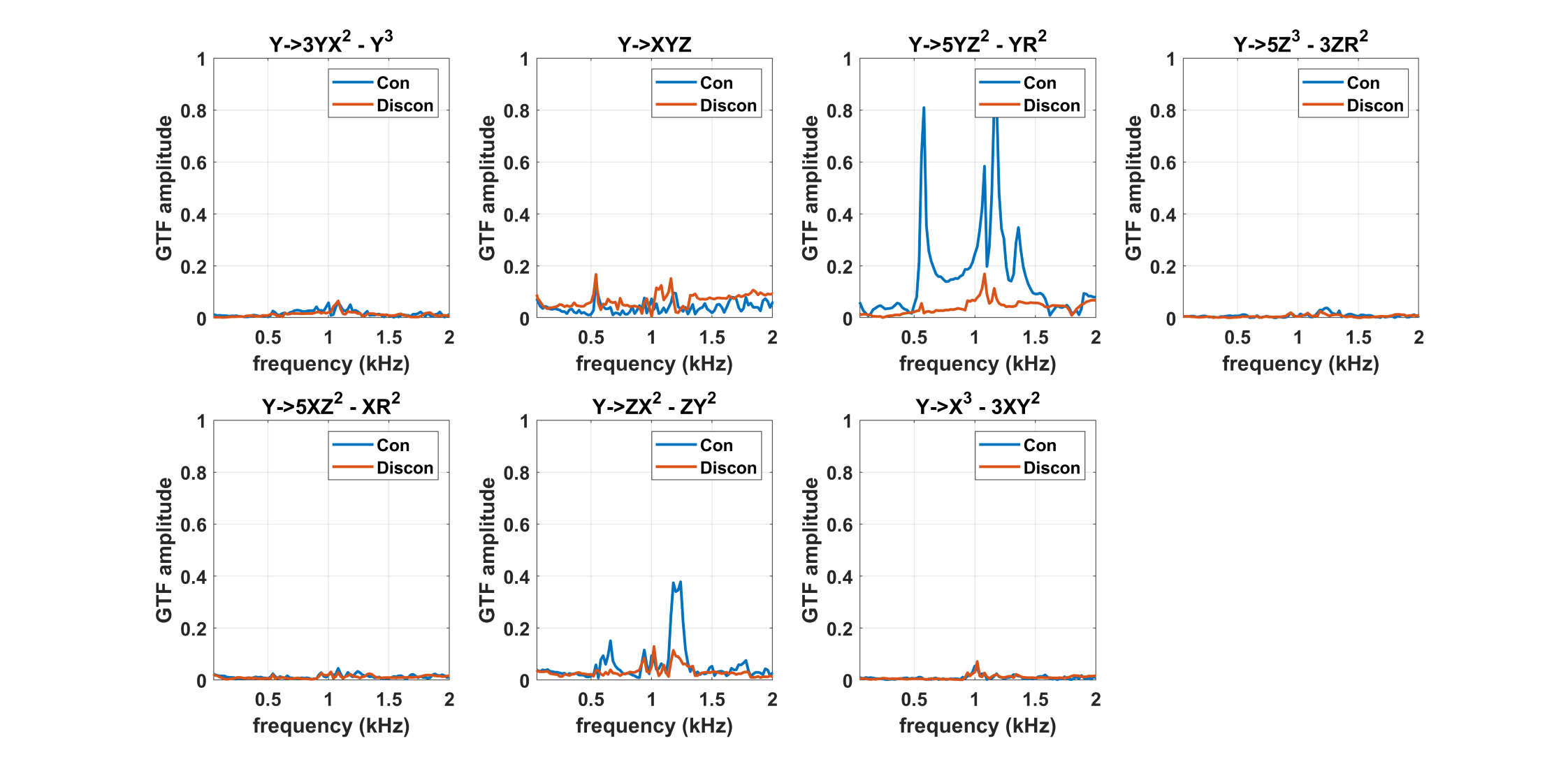
**

***Fig S3****. First to third order field cross-terms measured with a dynamic field camera at 11.7T with the 3^rd^ order shim coils connected and disconnected, when pulsing on the Y gradient axis.*

**
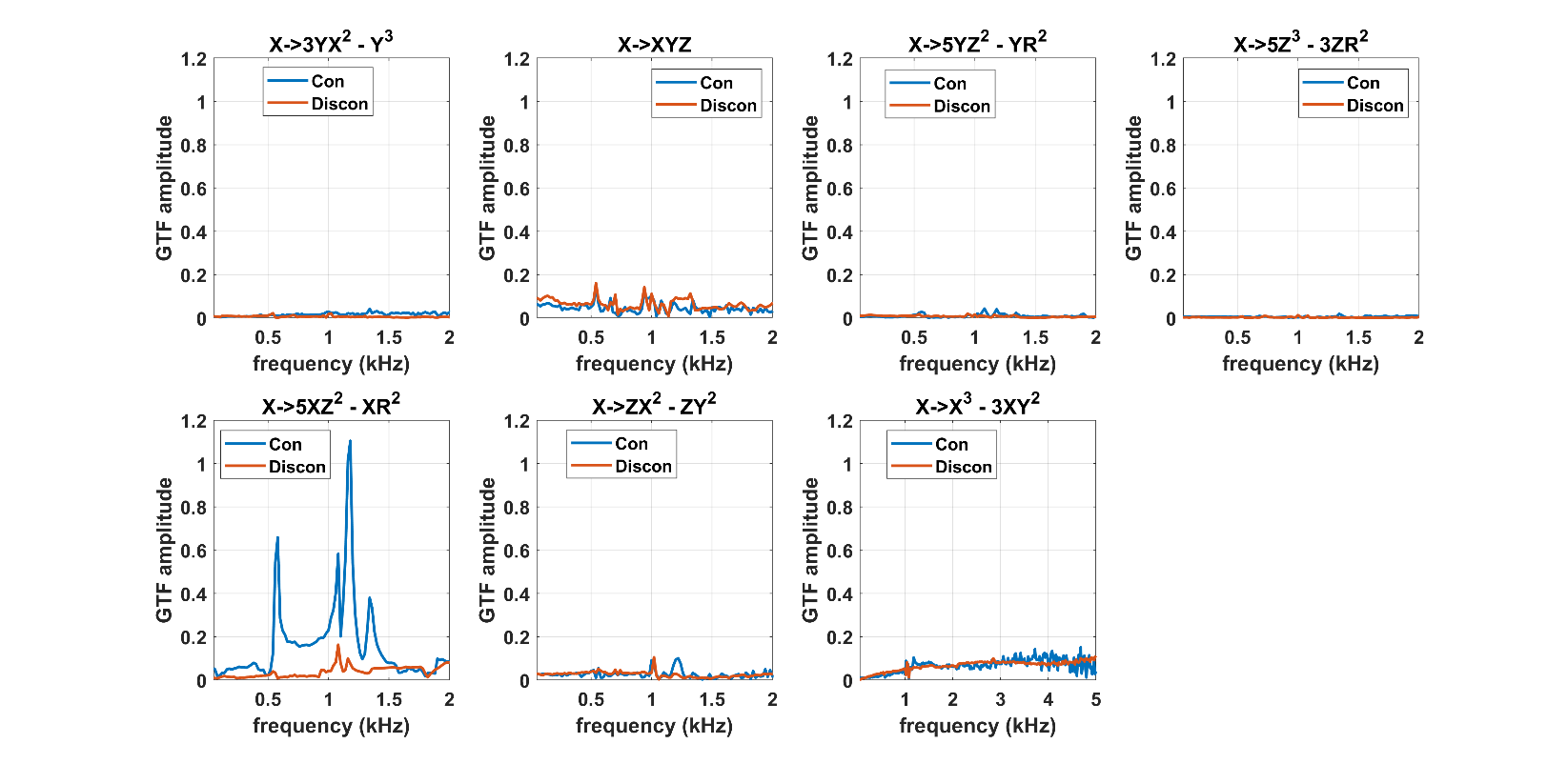
**

***Fig S4****. First to third order field cross-terms measured with a dynamic field camera at 11.7T with the 3^rd^ order shim coils connected and disconnected, when pulsing on the X gradient axis.*
